# Management of Children's Teeth

**Published:** 1887-09

**Authors:** J. E. Cravens

**Affiliations:** Indianapolis, Ind.


ARTICLE II.

### MANAGEMENT OF CHILDREN'S TEETH.

BY J. E. CRAVENS, D. D. S., INDIANAPOLIS, IND.

[Read before the Indiana State Society.]

This is one of the gravest problems that the dental practitioner is called upon to solve; and there is, perhaps, less chronicled information upon this than upon any other special division of dental practice.

It is not to be expected that this whole subject shall be embraced in a single paper, so that certain points about which the least has been written, will be presented for your consideration.

As a rule, children under three years of age are not brought to the dentist, therefore his ministrations begin, usually, with completely erupted sets of deciduous teeth. The family physician is presumed to exercise supreme supervision of the difficulties of primary dentition, in all subjects under the age of three years; only occasionally condescending to call the dentist into consultation as a source of much needed information. Whether or not the desired information is obtained, depends upon the dentist's opportunities for observation and his habit of improving or neglecting opportunities. In any event, the practitioner falls far short of what is rightfully expected of him by his profession, and of what is due to the little patients—whether expected or not, when he (the dentist) fails to comprehend the importance of retaining the deciduous teeth, and of teaching parents to properly appreciate them.

This instruction of parents is a labor of love and the limited number of dentists who engage in it, often find it unprofitable, thankless and discouraging. There are several reasons why the deciduous teeth should be retained until their successors are ready to erupt, and why they should receive thoughtful and skillful attention at the hands of the dentist.

The deciduous teeth are always regular in arrangement, showing them to be in nowise accidental True they are frequently tardy, but so are other organs. They never fail to come.

Let us consider the wisdom of the order of deciduous dentition from a chronological view. These teeth erupt in groups, and the presence of the several groups furnishes an index, more or less accurate, by which may be determined the approximate age of the individual, the stage of development of certain hidden organs, particularly the stomach and indicates fairly the character of food demanded for the further development of the individual. The index thus provided is lucid enough to be consulted with profit by any intelligent, observing dentist, or even by physicians. But the physician derives no benefit from this deciduous dental index, because "he ain't built that way," and that is why he occasionally calls the dentist in consultation.

In adult subjects the masseters are the strongest pair of muscles in the body, being capable of maintaining the suspended weight of the body, and often more; but in childhood the masseters do not possess this proportionate strength, so that often the mastication of solid or fibrous food would be for the young subject extremely difficult if not impossible, were it not for the fact that these teeth bear peculiarly sharp cusps for the quick and easy mastication of substances that otherwise would necessarily be bolted whole and uninsalivated into the stomach.

This facility of deciduous teeth for chopping the food meets the demand of stomachs of very limited digestive capability. In this respect the deciduous teeth that have been freshly erupted, present some analogy to the third stage of mechanical abrasion common to the advanced age of the human subject that has been characterized as second childhood, and in which the sharp edges of the abraded teeth enable the aged individual to comminute his food.

Every parent has probably noted the sharp spines that surmounted the cutting edges of the baby's new incisors. These spines possess wonderful penetrating power, but as the individual grows stronger, power comes to the masseters and the spines are soon worn away, leaving a plain incisive edge that is better adapted to the habits of the developing individual.

The adult teeth are from one-third to one half larger than the deciduous ones, and fifty per cent. more numerous, requiring about double the capacity of alveolar arch, which the child's jaw must grow to accommodate.

This additional development of the alveolar arch is usually accomplished in six years, beginning at about the sixth year. Yet how often do we find the inferior adult centrals, at six years, coming through the gum behind the deciduous incisors and obstructing the movements of the tongue. And a little later, six months perhaps, the inferior laterals struggle through, so far back and so hedged and handicapped for space and position as to *appear* to be hopelessly shut out of the arch forever.

Right here is where mistakes are often made by dentists, by prematurely extracting deciduous incisors. So long as deciduous teeth remain and in healthy condition, the arch may be expected to expand, at least until the requisite space for the accommodation of the adult teeth has been attained. The deciduous crowns and roots seem to serve as levers and wedges by which the jaw is induced to expand at the desired points. The tongue possibly is an active factor in rendering the leverage effective.

Another reason for retaining the deciduous teeth as long as space is needed, is that the resorption of their roots is attended by a mild form of local inflammation and increased vascularity, resulting in hypernutrition of the jaw, and consequent enlargement of the alveolar arch for the accommodation of adult teeth. When it is possible to do so, all cavities in deciduous teeth should be filled, otherwise they may become so extensively decayed as to necessitate extraction.

Pulpless deciduous teeth should be retained, if possible, under conditions that may not prevent resorption of their roots, to the end that they may perform their secondary function of assisting in inducing enlargement of the alveolar arch.

The deciduous molars should be retained for good mechanical reasons. They prevent a forward tipping of the sixth year molars, particularly of the lower ones, thus preserving proper space for their immediate successors, the bicuspids. Also, they are necessary to preserve articular surfaces for the effective mastication of food. Good masticating ability is quite as essential for the child as for the adult.—*Western Dental Journal.*

